# Differences between repeated lipid profile measurements in a tertiary hospital over a short time period

**DOI:** 10.1186/s12944-024-02022-2

**Published:** 2024-01-27

**Authors:** Or Porat, Marriele Kaplan, Smadar Atlibenkin, Dalia Hasson-Gilad, Amir Karban, Ronen Zalts

**Affiliations:** 1 The Ruth and Bruce Rappaport Faculty of Medicine, Technion - Israel Institute of Technology, Haifa, Israel; 2 Biochemistry Laboratory, Rambam Health Care Campus, Haifa, Israel; 3 Internal Medicine Department C, Rambam Health Care, Campus, Ha’aliah street 8, Box 9602, Haifa, 3109601 Israel

**Keywords:** Low-density lipoprotein cholesterol, LDL-C calculation, Lipid profile, Fasting, Repeated tests

## Abstract

**Background:**

Measurement of the plasma lipid profile, mainly low-density lipoprotein cholesterol (LDL-C), is widely used in the management of hospitalized patients as part of their cardiometabolic risk assessment. In common practice, LDL-C is calculated indirectly by the Friedewald equation. For many years, fasting of 8–14 h is needed to obtain an accurate lipid profile measurement, although recent guidelines do not necessitate it. The aim of this study was to find patients with two consecutive LDL-C measurements taken over a short time period on the same admission to see if a significant difference exists and to suggest reasons that may explain it. We also aim to define whether the difference between LDL-C calculated by the Friedewald equation is diminished while using the newer Martin/Hopkins, de Cordova or Sampson/NIH equations.

**Methods:**

This was a retrospective cohort study performed in one medical center in Israel. In a five-year time period, 772 patients with two repeated LDL-C measurements taken on the same admission were found. The median time gap between tests was 2 days. Correlations between laboratory results and LDL-C measurements were determined.

**Results:**

A total of 414 patients (53.6%) had a difference greater than the acceptable total error of 8.9% in LDL-C calculation using the Friedewald equation, with a mean 25.8% difference between the two tests. Newer LDL-C calculations showed less diversity. Non-HDL-C was found as the only variable with a major correlation with LDL-C results in all equations. A weaker correlation was found with HDL-C. Triglycerides showed an even weaker correlation, and glucose differences had no correlation with LDL-C differences.

**Conclusions:**

Repeated LDL-C measurements can vary widely, even during a short period of hospitalization. In this study, more than half of the patients had a significant difference between their consecutive LDL-C results. This wide difference between two consecutive tests was diminished using newer calculations, yet not well explained. The fasting state likely has no effect on LDL-C levels. The results of this study might emphasize that many factors influence LDL-C calculation, especially in the disease state. Further research is needed, especially in looking for a more accurate LDL-C calculation from existing formulas.

## Background

Measurement of the plasma lipid profile is widely used in the management of hospitalized patients as part of their cardiometabolic risk assessment [1]. Low-density lipoprotein-cholesterol (LDL-C) is considered to be the most important measurement within the lipid profile due to its central role in atherogenesis and serves as the main therapeutic target in primary and secondary atherosclerotic cardiovascular disease (ASCVD) prevention [2]. However, in common practice, LDL-C is not measured directly but rather calculated indirectly using the laboratory measures of total cholesterol (TC), high-density lipoprotein-cholesterol (HDL-C) and triglycerides (TG). The most commonly used calculation of LDL-C is the equation introduced in 1972 by Friedewald et al. [3], in which LDL-C is calculated by (total cholesterol) − (HDL-C) − (TG/5). All values are expressed in mg/dL. The TG/5 term serves as an estimation of very low-density lipoprotein-cholesterol (VLDL-C), but its accuracy diminishes at TG levels over 177 mg/dL [4]. For TG levels over 400 mg/dl, LDL-C cannot be calculated by this equation.

To overcome the miscalculation of VLDL-C in Friedewald’s equation, several newer LDL-C calculations have been proposed. At least three of these calculations are based on large-scale studies. The Martin/Hopkins equation, introduced in 2013, replaces the fixed TG denominator of 5 in the Friedewald equation with an empirical factor that varies depending on levels of TGs and non–HDL-C [5]. In the same year, a simpler calculation of LDL-C was introduced by de Cordova et al. [6]. A more recent calculation, the Sampson/NIH equation, was developed for patients with TG levels up to 800 mg/dl and showed a more accurate calculation of LDL-C, also in patients with low LDL-C [7]. However, none of these calculations are in widespread use, mainly because of insufficient validation, and the Friedewald equation continues to serve as the main estimation of LDL-C.

In most health care centers in the world, fasting of 8 to 14 h is needed from adult patients before the lipid screening test is drawn [8]. However, data from large-scale studies suggest that fasting has little effect on the lipid profile and probably better reflects the true ASCVD risk [8–11]. According to the guidelines of the European Society of Cardiology from 2019, which are based on these studies, fasting is not necessary before lipid profile screening tests [2]. In patients with familial hyperlipidemia, hypertriglyceridemia or metabolic syndrome, fasting has a greater effect on the TG level, which considerably affects the level of the calculated LDL-C. In this group of patients, fasting is indeed recommended. The main limitation of these studies is that comparisons were made between large-scale fasting and nonfasting blood samples and not between two fasting and nonfasting samples of the same individual. Assuming there is indeed no necessity for long fasting, two lipid profile tests taken from one patient at different times since last meal, should show similar results. The acceptable difference between two tests in the same laboratory taken at the same time is up to 8.9% for TC and HDL-C and up to 14.9% for TG [12].

The aim of this study was to determine whether there is a difference in LDL-C in patients in whom lipid profile measurement was repeated at the same admission over a time period of no more than five days. This time period might diminish the effect of other factors, such as acute illness, lipid lowering agents, drug interactions and other probable confounders. If a difference does exist, suggest reasons that may explain the difference. We also aim to define whether the difference between LDL-C calculated by the commonly used Friedewald’s equation is diminished while using the newer equations, which demonstrated a more accurate LDL-C calculation.

## Materials and methods

### Study population and data collection

We conducted a retrospective study of patients admitted to the internal medicine division in Rambam Health Care Campus, a tertiary hospital in northern Israel, between 01/01/2015 and 31/12/2019. The data were collected from computerized medical records of the hospital. We surveyed patients with multiple lipid profile tests taken during their hospitalization. Usually, there is no indication for repeated cholesterol measurements during the same admission; our assumption is that most of them were taken by mistake or by physician decision to repeat the lipid profile on different fasting conditions. The policy of the biochemistry laboratory in our hospital is to perform all submitted tests and not to cancel repeated tests. We excluded patients with missing HDL-C results and patients with TG measurements > 400 mg/dl on one or more lipid profile measurements. Patients with a gap of six or more days between the two measurements were excluded. The data collection included demographic variables, clinical variables such as ischemic heart disease, diabetes mellitus and hypertension, laboratory results including lipid profile, glucose, creatinine, liver enzymes, hemoglobin and TSH levels, and usage of lipid lowering agents.

### Cholesterol and triglyceride measurements

The cholesterol assay was performed in an automated clinical chemistry enzymatic assay. HDL cholesterol was measured in a method based on accelerating the reaction of cholesterol oxidase with non-HDL unesterified cholesterol and dissolving HDL cholesterol selectively using a specific detergent. Triglycerides were measured with enzymatic hydrolyzation based on the glycerol phosphate oxidase reaction. All measurements were performed with an Abbott kit on the ARCHITECT-c systems.

### Calculation of LDL-C

Four fourmulas to calculate LDL-C were used:

1. The Friedwald equation, in which LDL-C = (total cholesterol) − (HDL-C) − (TG/5) [3].
2. The Martin/Hopkins equation, in which LDL-C = (total cholesterol) − (HDL-C) − (TG/empirical factor) [5].
3. de Cordova equation, in which LDL-C = 0.75(TC - HDL-C) [6].
4. Sampson/NIH equation, in which LDL-C = TC/0.948 – HDL-C/0.971- (TG/8.56 + TG*non-HDL-C/2140 – TG^2^/16,100) – 9.44 [7].

### Statistical analysis

Normal distribution was assessed using Skewness and Kurtosis. The distribution of the demographic and laboratory results was presented in terms of means and standard deviations (SD). For each of the indices, the difference between the first and second measurements was calculated as the “percent of change”. Correlations between variables were calculated using Pearson *r* correlation coefficients. Comparisons between matched pairs of variables, such as the four different LDL-C calculations, were executed using paired-samples t-tests. Differences between groups of patients (such as gender) were executed using independent samples tests. Additionally, indices were split into three levels: below the normal range, within the normal range and above the normal range. Differences in the first and second measurements and the percent of change by these three level variables were executed using one-way ANOVA (analysis of variance), and the interaction between these three levels and patient gender was executed using two-way ANOVA. Additionally, partial eta squared was used to estimate the effect sizes of the differences.

## Results

**Fig. 1:**
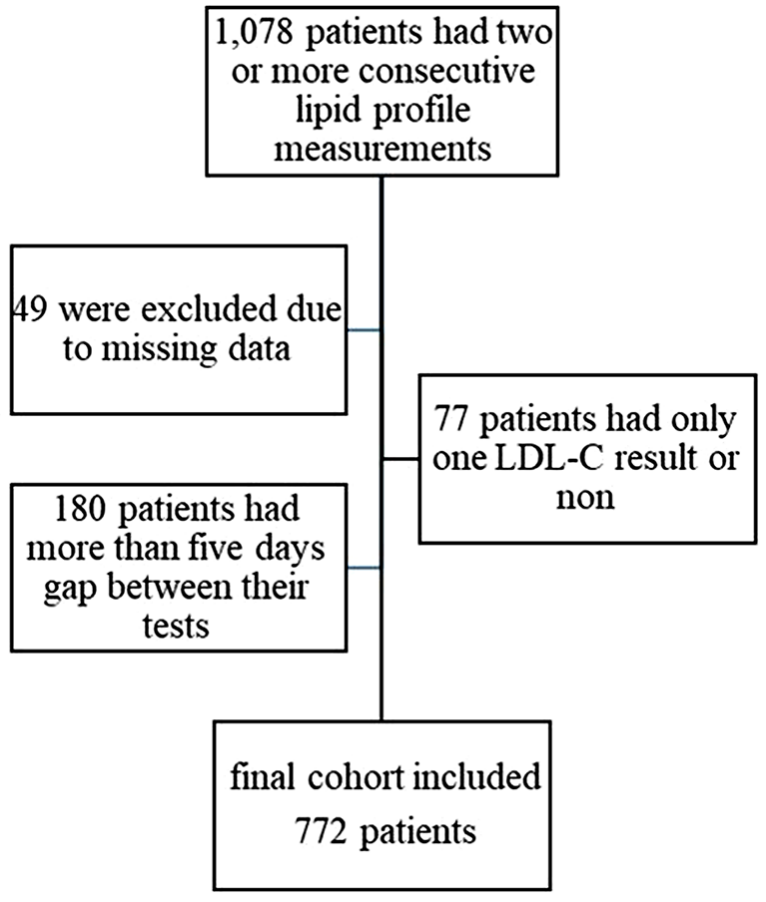
Patients recruited for the study

In this five-year time period, one thousand seventy-eight patients with two or more consecutive lipid profile measurements were found. A total of 958 had two repeated exams, 81 had three, 24 had four, and 15 had five or more on the same admission. After exclusion of patients with missing data, 1029 patients were eligible. Of the 1029 patients, 952 had two measurable LDL-C measurements, 59 had no LDL-C measurement at one of the two examinations, and in 18, both measurements were missing due to high triglyceride levels. After excluding patients who had a more than five-day gap between their lipid profile tests, the final cohort included 772 patients (Fig. 1).

The baseline characteristics of the patients included in the study and the mean lipid profile measurement results of the two tests are summarized in Table 1.

**Table 1:** Characteristics of the patients

Mean age ± SD;	58.4 ± 17.6
Male sex – no. (%)	484 (62.7%)
**Coexisting illness – no. (%)**	
Ischemic heart disease	236 (30.7%)
Diabetes mellitus	288 (37.5%)
Hypertension	431 (56.1%)
Smoking	331 (43.1%)
Days between tests – mean ± SD; (Median)	1.8 ± 1.3 (2.0)
**Lipid profile – mean ± SD, mg/dL**	
TC 1	159.5 ± 47.5;
TC 2	157.1 ± 45.3;
HDL 1	37.5 ± 16.1;
HDL 2	35.8 ± 15.1;
TG 1	137.4 ± 71.6;
TG 2	138.1 ± 67.9;
LDL (F) 1	94.4 ± 39.0;
LDL (F) 2	93.7 ± 37.5;
LDL (M) 1	98.1 ± 38.2;
LDL (M) 2	97.5 ± 36.6;
LDL (D) 1	91.9 ± 32.3;
LDL (D) 2	91.5 ± 30.7;
LDL (S) 1	97.1 ± 39.0;
LDL (S) 2	96.5 ± 37.4;

SD = standard deviation; TC = total cholesterol; LDL = low density lipoprotein, (F) = Friedewald equation, (M) = Martin/Hopkins equation, (D) = de Cordova equation, (S) = Sampson/NIH equation; HDL = high density lipoprotein; TG = triglycerides; 1 = first measurement; 2 = second measurement

**Fig. 2:**
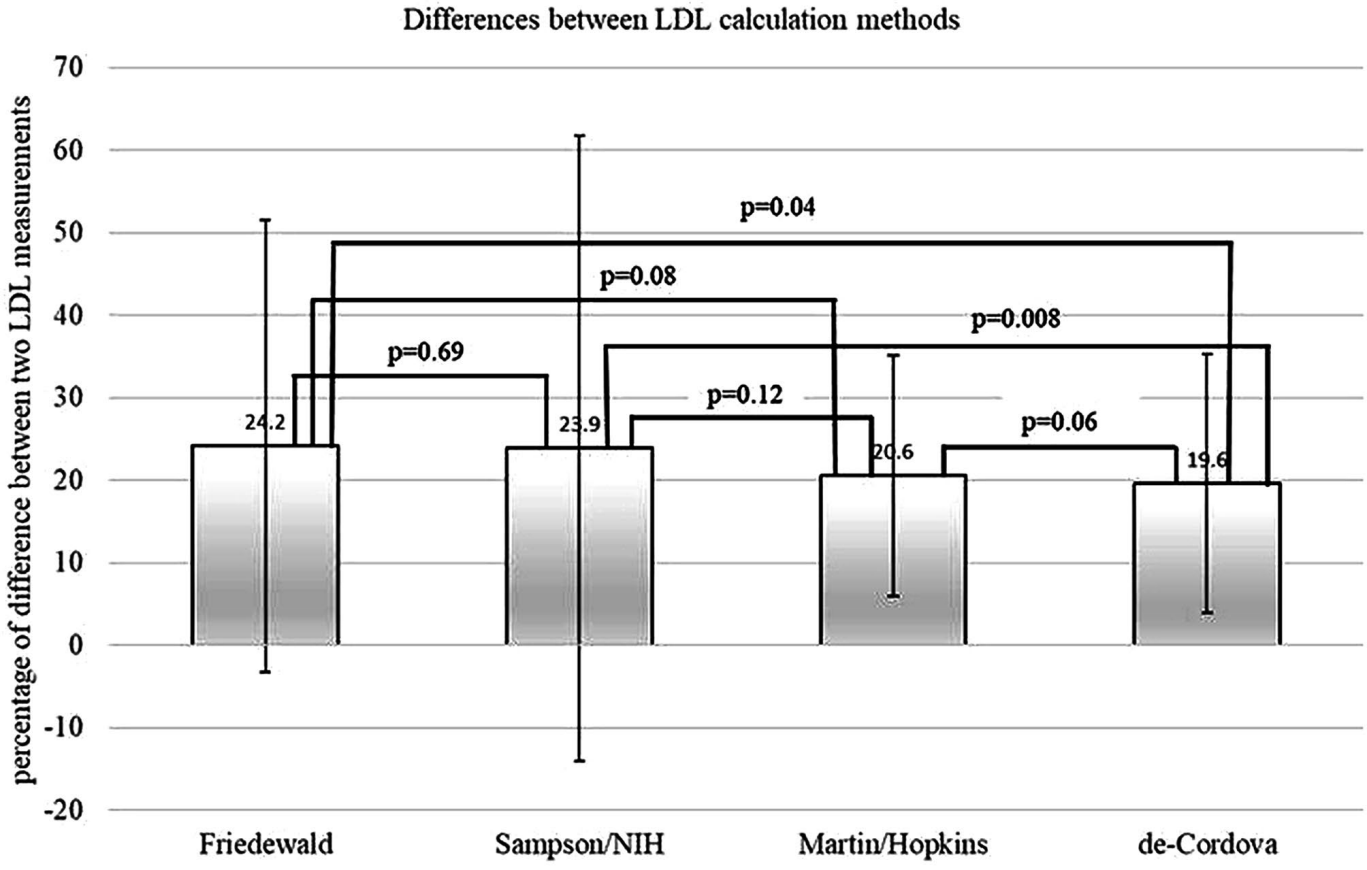
Means and standard deviations of the percentage of difference between two consecutive LDL-C measurements using different LDL calculation methods. Patients with a difference of more than 8.9% were included in this calculation

A total of 414 of the 772 patients (53.6%) had a difference greater than 8.9% in the Friedewald LDL-C calculation. A total of 172 patients (22.3%) had a difference greater than 20%, and 84 patients (10.9%) had a difference greater than 30%. Out of the 414 patients with a difference greater than 8.9% for LDL-C results, 236 patients (57.0%) had a difference greater than 8.9% in their total cholesterol results, and 250 patients (60.4%) had a difference greater than the acceptable for triglycerides (> 14.9%). Calculating LDL-C with the Sampson/NIH equation, 394 patients (51.0%) had a difference greater than 8.9%, 151 (19.6%) had a difference greater than 20%, and 67 (8.7%) had a difference greater than 30%. By calculating LDL-C with the Martin/Hopkins equation, 375 patients (48.6%) had a difference greater than 8.9%, 141 patients (18.3%) had a difference of 20% or more, and 55 (7.1%) had a difference of 30% or more. Using the de-Cordova equation, 332 patients (43.0%) had a difference greater than 8.9%, 105 patients (13.6%) had a difference greater than 20%, and 37 (4.8%) had a difference of 30% or more. The difference of more than 8.9% between two consecutive LDL-C measurements was statistically significant lower by using the de-Cordova equation (Fig. 2).

**Fig. 3:**
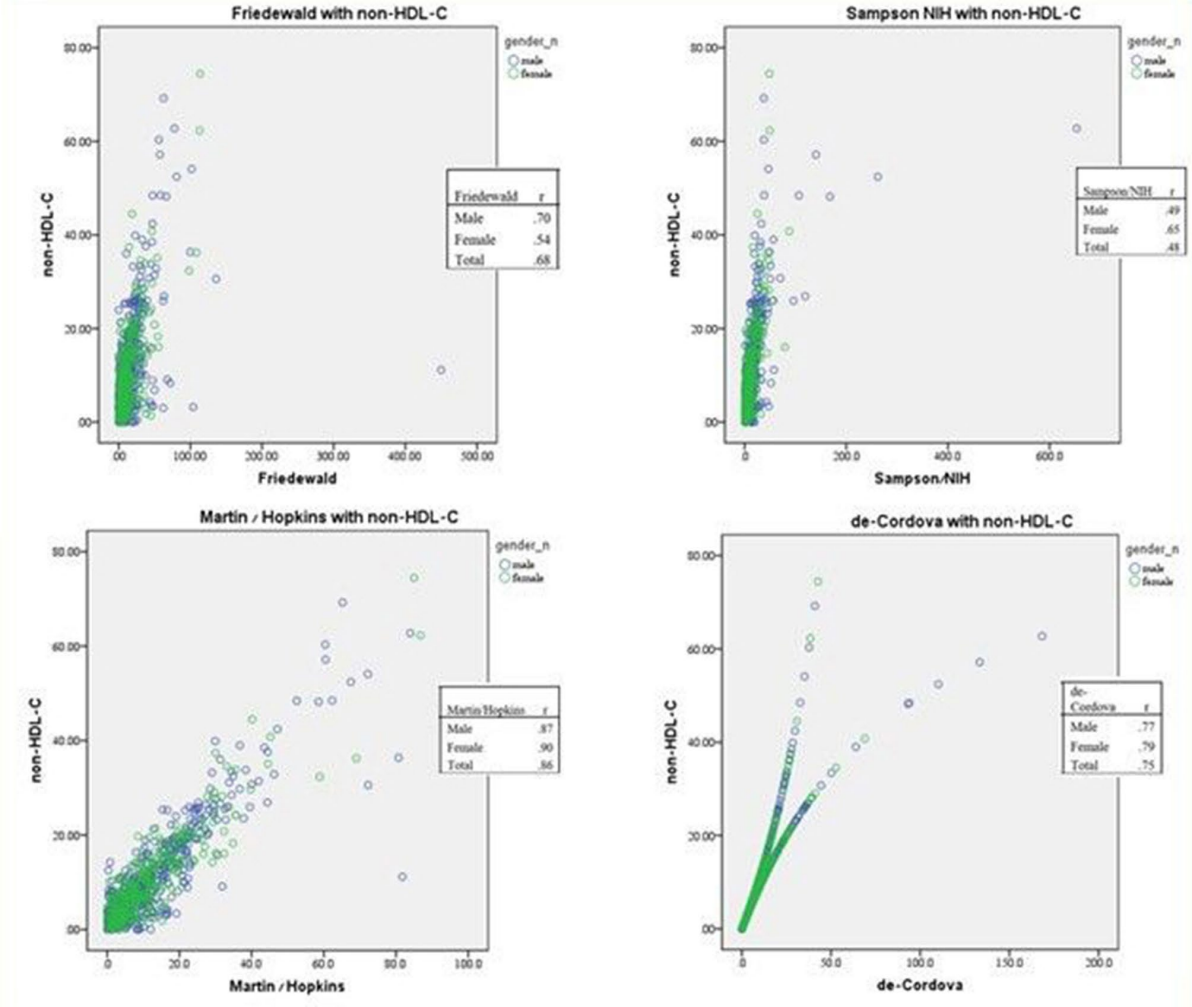
Scatterplots presenting the correlation of non-HDL-C and LDL-C calculated by the four equations

To determine which of the variables tend to affect the LDL-C differences, we examined their correlations with other laboratory result differences of the cohort. The group of patients with LDL-C differences of more than 8.9% was investigated separately for each calculation method. The difference between consecutive non-HDL results showed the strongest correlation with the LDL-C difference (*r* = 0.68, *P* ≤ 0.001). HDL-C differences showed a weaker correlation with LDL-C differences (*r* = 0.46, *P* ≤ 0.001). TG differences showed a weak correlation with LDL-C differences (*r*=-0.15, *P* ≤ 0.01). The glucose difference between the two tests showed no correlation with the LDL-C difference (*r* = 0.09, *P* > 0.05). However, in females, a moderate correlation was observed between glucose and LDL-C differences (*r* = 0.28, *P* ≤ 0.01). Other factors we investigated were the time gap between the two consecutive tests and other laboratory results: creatinine and liver enzyme (AST, ALT, ALP and GGT) differences. No remarkable correlations were found between these parameters and LDL-C differences. Additionally, no significant correlations were found related to patients’ medical background: ischemic heart disease, smoking, diabetes mellitus and hypertension. After calculating LDL-C with the other three calculation methods, the correlations were quite similar: LDL-C calculation differences were strongly correlated with non-HDL-C differences, less strongly correlated with HDL-C and almost not correlated with TG and glucose differences. These findings are summarized in Table 2. The correlation between non-HDL-C and LDL differences using the four equations is shown on Fig. 3.

**Table 2:** Pearson (*r*) intercorrelations between repeated non-HDL-C, HDL-C, TG and glucose and the four LDL-C calculations

Calculation method	Non-HDL-C			HDL-C			TG			Glucose		
	M	F	T	M	F	T	M	F	T	M	F	T
Friedewald	0.70^****^	0.54^****^	0.68^****^	0.53^****^	0.20^**^	0.46^****^	0.15^**^	0.17^**^	0.15^***^	0.08^*^	0.28^**^	0.09^*^
Sampson/NIH	0.49^****^	0.65^****^	0.48^****^	0.22^****^	0.25^***^	0.22^****^	0.07^*^	0.26^***^	0.09^*^	0.11^*^	0.11^*^	0.07^*^
Martin/Hopkins	0.87^****^	0.90^****^	0.86^****^	0.39^****^	0.25^***^	0.35^****^	0.10^*^	0.12^*^	0.10^**^	0.05^*^	0.39^****^	0.09^*^
de-Cordova	0.77^****^	0.79^****^	0.75^****^	0.34^****^	0.23^***^	0.33^****^	0.14^**^	0.02^*^	0.11^**^	0.13^*^	0.07^*^	0.05^*^

HDL-high-density lipoprotein; TG = triglycerides; M = male; F = female; T = total

The numbers denote the Pearson intercorrelations. *r* = 1.0 means a strong correlation; *r* = 0.0 means no correlation

*P = nonsignificant, ^**^P ≤ 0.05, ^***^P ≤ 0.01, ^****^P ≤ 0.001

## Discussion

The major finding of this study is that repeated cholesterol tests can vary widely, even during short periods of hospitalization. More than half of the patients in our cohort had a significant difference between their consecutive LDL-C results (greater than 8.9%). A possible explanation for the variation in consecutive LDL-C results could be the fact that medical conditions have a great influence on the lipid profile during hospitalization. Previously published studies have found that common medical procedures such as angioplasty, coronary arteriography and general surgery or even minor illnesses such as upper respiratory tract infections can increase or decrease the lipid profile results in less than 24 h [13–16].

Although the median time gap between two consecutive tests performed on the same patient was two days, wide variation was still found. To explain this difference, we tried to correlate it with other possible factors. One major factor that might explain this is fasting status. The data about this status could not be retrieved retrospectively from the patients’ charts, thus serving as a major limitation of this study. However, assuming that glucose and TG level differences can estimate fasting or nonfasting states, correlations were performed between these values and LDL-C. A very weak correlation was found between TG and LDL-C levels, and a weaker correlation was found between glucose and LDL-C, thus reinforcing professional associations’ guidelines and expert panels that prior fasting is unnecessary for lipid profile tests [17–21]. Nonfasting lipid measurement tests can simplify the procedure for the patient, minimize the risk for hypoglycemia in diabetic patients and eliminate the need for repeated tests [22].

Another aim of this study was to use our cohort of repeated LDL-C measurements as a tool to compare the commonly used Friedewald’s equation with three of the newer LDL-C calculation methods, and by this try to locate which demonstrates a more accurate LDL-C calculation. We found that the Friedewald calculation showed the highest percentage of difference between two consecutive tests, with an average 24.2% difference between tests. The calculation that showed the lowest percentage of difference was the de-Cordova equation, with a 19.6% difference and the lowest standard deviation. This finding suggests that the de-Cordova equation implies a more accurate calculation of LDL-C. All LDL-C calculations showed similar strong correlations to non-HDL cholesterol, thus validating the relationship between LDL-C and non-HDL-C. Similarly, all equations showed no correlation to glucose difference and TG difference, thus strengthening the assumption that LDL-C is not influenced by fasting state.

There is no clear recommendation about the measurement of lipid profiles during hospitalization. The National Cholesterol Education Program (NCEP) recommends that lipid profiles should be measured in all patients with chest pain or acute coronary syndrome, preferably in the first 24 h of hospital admission [8]. Repeated lipid profile tests during hospitalization are probably not necessary. Repeated tests are probably common in health care centers worldwide, but the extent of this problem remains almost unreported [23]. The wide difference between two consecutive lipid profile tests found in this study might emphasize that many factors influence LDL-C calculation, especially in the disease state.

### What does the current study add to the existing knowledge?

This study focuses on significant differences in LDL-C values between two consecutive lipid profile measurements for the same patient performed on admission. Logically, there is no reason for the major difference in LDL-C measurement, yet in more than half of the cases, a significant difference was found. The other three calculations tested in this study still varied in both measurements but showed significantly less difference.

### Study strengths

To the best of our knowledge, this is the first study that investigates repeated lipid profile tests performed for the same patient over a short period of time on the same admission. In most hospitalized patients, there is no need to measure lipid profile, and certainly there is no clinical reason to repeat it within the same admission. Most of these tests were probably performed unintentionally. Over a 5-year time period, a large number of repeated tests were collected, thus providing good statistical power.

### Study limitations

The main limitation of this study is its retrospective, observational design. To assess the possible causes for different LDL-C levels between two consecutive blood tests, a large-scale prospective study should be performed. Another limitation is the lack of information about the fasting state of the subjects. We assumed that large differences in glucose and TG levels can assess fasting, but of course, this is just a speculation. Many factors that can influence the lipid profile could not be taken into account, although exclusion of patients with a gap of more than five days might reduce these confounders. Finally, LDL-C was calculated based on the Friedewald equation, based on the estimation that VLDL-C is calculated by the ratio TG/5. We have no direct calculation of LDL-C in the current study. A recent review and meta-analysis analyzing the best method to calculate LDL-C in clinical practice concluded that further validation of the different equations is needed in different populations [24].

## Conclusions

LDL-C levels are probably influenced by multiple factors, and their values can vary widely, even in a short time period on the same admission. The fasting state probably has little effect on LDL-C measurement, as supported by other studies. The de-Cordova calculation of LDL-C seems to have less variety than other calculations, and the commonly used Friedewald calculation shows the largest difference between two tests in the same patient. Further research is needed, especially in looking for a more accurate LDL-C calculation from existing formulas. Prospective comparison of direct LDL-C measurement to different LDL-C calculations might be considered.

## Data Availability

The datasets used and/or analyzed during the current study are available from the corresponding author upon reasonable request.

## Abbreviations

LDL-C: Low-density lipoprotein-cholesterol
HDL-C: High-density lipoprotein-cholesterol
TC: Total cholesterol
TG: Triglycerides
VLDL-C: Very low-density lipoprotein-cholesterol
ASCVD: Atherosclerotic cardiovascular disease
SD: Standard deviation
IQR: Interquartile range
